# Feasibility of OptiMaL, a Self-Management Programme for Oesophageal Cancer Survivors

**DOI:** 10.1177/10732748231185002

**Published:** 2023-08-24

**Authors:** Eilish King, Naomi Algeo, Deirdre Connolly

**Affiliations:** 1 Discipline of Occupational Therapy, 155276 School of Medicine, Trinity College, Dublin, Ireland

**Keywords:** cancer survival, oesophageal cancer, quality of life, self-management, occupational therapy

## Abstract

**Introduction:**

There is limited availability of self-management interventions for oesophageal cancer survivors at present. This study examined the feasibility of OptiMal, a six-week, self-management programme to improve fatigue, mood and health-related quality of life for oesophageal cancer survivors.

**Methods:**

A mixed methods design was used to evaluate the feasibility of OptiMal. The quantitative arm of the study examined changes in the Multidimensional Fatigue Inventory, Hospital Anxiety and Depression Scale, and the EQ-5D-3L, administered prior to OptiMal (T1), immediately following completion of OptiMal (T2), and three months following completion (T3). Qualitative inquiry in the study was guided by a qualitative descriptive approach through focus groups investigating the experiences of group participants, and individual semi-structured interviews at T3. Qualitative data were analysed using thematic analysis.

**Results:**

Two OptiMal programmes were delivered over a six-month period with a total of fourteen individuals who had finished treatment for oesophageal cancer. The attendance rate was 89.3%. Statistically significant reductions were observed in fatigue, difficulty performing usual activities, anxiety and depression at three-month follow-up. Qualitative findings identified acceptability of the content and delivery format of OptiMal. Participants reported applying self-management strategies acquired through OptiMal to increase participation in daily activities and improve their health and well-being.

**Conclusions:**

This feasibility study yielded promising results in terms of self-management outcomes for oesophageal cancer survivors following attendance of OptiMal. Larger scale research studies with control groups are warranted to examine the outcomes in a robust manner.

## Introduction

Oesophageal cancer presents a variety of complications for health, well-being and participation in daily life activities. The prevalence of oesophageal cancer appears to be growing, with reports estimating it to be the sixth most common cause of death by cancer.^1^ In Ireland, 6706 cases of oesophageal cancer were reported between 2000 and 2017.^2^ Treatment for oesophageal cancer often involves surgical intervention, along with chemotherapy and radiation therapy.^3^ Oesophageal cancer has been associated with slow recovery and poor prognosis.^4^ Many oesophageal cancer survivors experience post-operative complications, with longer-term impact on fatigue and energy levels, mental health and participation in daily life.^5^ As more individuals are living with and beyond oesophageal cancer, it is important to support patients with managing persistent effects of cancer and treatment and to participate in daily life. In line with a survivorship approach, there is increasing recognition that oesophageal cancer survivors can benefit from supports following surgery to transition to life post-treatment and build a satisfying daily routine.^1^

Post-operative complications and persistent symptoms such as fatigue and distress are common following completion of oesophageal cancer treatment. Fatigue is frequently reported with 41–60% of oesophageal cancer survivors experiencing significant fatigue in the post-surgical period, persisting for over 12 months for many survivors.^6–8^ The impact of fatigue may decrease over time, Djärv and colleagues (2008) found that participants reported reduced fatigue three years post-surgery, when compared to 6 months post-surgery.^9^ Tsou and colleagues outlined that fatigue is often one of the most significant and poorly controlled symptoms of cancer, or cancer treatment, and that it often results in other difficulties such as disturbed sleep, reduced attention and other functional impairments.^6^ Schandl and colleagues reported that oesophageal cancer survivors experienced a wide variety of difficulties related to eating, nutrition and digestion in the year following oesophageal cancer surgery.^8^ Several studies have reported that reflux can persist for over 1 year post-surgery, along with symptoms such as dry mouth and dyspnoea,^10^ qualitative studies describe difficulties with eating following surgery, issues with constipation or diarrhoea, and weight loss, nausea, bloating or digestive difficulties.^11,12^

Anxiety and/or depression appear to be common amongst oesophageal cancer survivors, particularly within 1 year of diagnosis and treatment.^13^ The incidence of anxiety and depression appears to peak shortly after completion of surgery, with studies reporting between 40% and 63.3% of participants meeting the criteria for probable anxiety or depression.^14,15^ Schandl et al reported that 11% of their participants reported clinically significant symptoms of anxiety, and 12% of participants reported clinically significant symptoms of depression in the year following surgery.^8^ However, Liu and colleagues reported clinically significant symptoms of anxiety or depression in up to 23.3% of their participants in their study of psychological distress and Health-Related Quality of Life (HRQoL) 2 years post-surgery.^16^

Psychological distress and decreased energy levels have a significant impact on the health and well-being of oesophageal cancer survivors, and have been cited as predictors of poorer survival^13,16,17^ following treatment. Qualitative studies lend support to these findings, describing the impact of psychological distress in the recovery period from oesophageal cancer, with many participants identifying the impact of fear of cancer recurrence and pain during post-operative period.^11^

Survivorship supports for oesophageal cancer survivors are continuing to emerge. A nursing led intervention that included patient education, health management and healthy lifestyle habits, reported positive outcomes for self-care ability, social relationships, and anxiety and depression amongst those who participated in the intervention when compared to a control group.^18^ An exercise intervention with a 12-week brisk walking programme and dietary education yielded positive outcomes for reflux symptoms, anxiety and sleep quality.^19^ A nursing led series of education and counselling sessions that focused on nutrition for oesophageal cancer survivors in the post-surgical period was helpful in enabling patients to understand physical changes following treatment and managing nutrition.^20^ Currently, support programmes that cater for oesophageal cancer survivors in the post-treatment period tend to target specific topics such as nutrition or exercise. However, there is limited evidence of self-management programmes to support oesophageal cancer survivors to participate in meaningful daily life activities, and to manage persistent symptoms or effects of oesophageal cancer.

OptiMal is a group-based, self-management intervention that focuses on providing individuals with skills and confidence to manage persistent symptoms of fatigue and distress and facilitate participation in daily activities. It was initially developed for people with multimorbidity and then adapted for use with women with breast cancer.^21–24^ The purpose of this study therefore, was to explore the feasibility of OptiMal for oesophageal cancer survivors. The specific objectives included:

• To examine the feasibility of OptiMal as a multidisciplinary self-management intervention for individuals with oesophageal cancer
• To examine participant-reported changes in fatigue, anxiety, depression and HRQoL following participation in OptiMal
• To explore participants' experiences and acceptability of OptiMal
• To explore application of knowledge of symptom management from OptiMal into daily activities following completion of the programme

## Materials and Methods

### Intervention

OptiMal is based on self-management support theory to increase individuals’ knowledge and skills to manage long-term health conditions. It is underpinned by Bandura’s theory of self-efficacy. The original OptiMal programme was designed to meet the needs of people with multimorbidity within a primary care context.^21^ OptiMal is facilitated by an occupational therapist with physiotherapy and dietician input on physical activity and nutrition.

**Figure 1.**
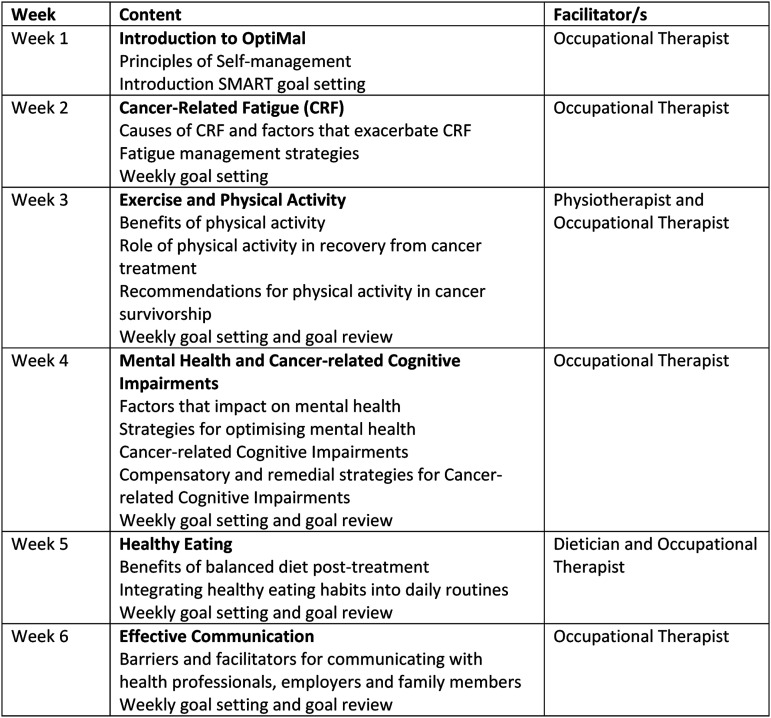
Content and implementation process of OptiMal.

The programme consists of 6 group-based sessions delivered in-person over the course of 6 weeks for 2.5 hours/session. Programme content focuses on management of persistent symptoms commonly experienced by individuals with oesophageal cancer. These include fatigue, distress, reduced exercise tolerance and difficulty with weight management. Figure 1 outlines details of weekly content of OptiMal. Each session is structured to include education on the weekly topic, peer-discussion and sharing of self-management strategies. Previous research of OptiMal recommended a maximum of 6-8 participants in order to facilitate group discussion.^22^ Each week also includes goal-setting activities to enable participants to implement learning during and after completion of the programme. Lorig et al.^25^ recommend weekly goal-setting to facilitate effective application and sustainability of self-management skills. All participants receive a handbook that contains information on each of the weekly topics, a copy of the slides presented each week and goal-setting templates. OptiMal was delivered in the academic institution in which the authors are located.

Two OptiMal programmes were delivered during the study period. Fidelity of the intervention was monitored by one of the authors (DC), who is an occupational therapist, and who facilitated both programmes. A weekly log was completed to record changes to content and/or delivery format following each session. DC was also present at the physical activity and nutrition sessions to note any changes to intervention content and delivery. The same physiotherapist and dietician delivered the physical activity and nutrition sessions in both OptiMal programmes delivered over the study period. There were no significant changes to content and/or delivery format across the two programmes.

### Study Design

This feasibility study utilised a mixed methods design. The quantitative phase was a pre-test, post-test design, while the qualitative phase used a qualitative descriptive design as outlined by Sandelowski.^26^ The research was guided by the Medical Research Council Framework for complex intervention research, which is concerned with examining whether interventions are generalisable, and feasible to implement.^27^ This study examined the feasibility and acceptability of OptiMal, in order to inform the next stage of evaluation. The reporting of this study conforms to COREQ guidelines^28^ for qualitative studies and CONSORT statement extension for feasibility studies.^29^

### Participant Recruitment

Billingham et al.,^30^ stated that although a sample size justification is important for feasibility and pilot trials, a formal sample size calculation may not always be appropriate. OptiMal had previously been tested for individuals with different types of cancer,^22^ however, that study did not include individuals with oesophageal cancer who have unique needs regarding self-management of post-treatment symptoms.^4^ Therefore, as the primary purpose of this study was to examine the acceptability of OptiMal for people with oesophageal cancer, a sample size was not calculated.

Inclusion criteria for this study were individuals aged over 18 years of age, who had completed treatment for oesophageal cancer. Participants self-selected for this study based on self-identified need for education and support to manage post-treatment difficulties. Information regarding OptiMal, and the aims of the study, was presented at an oesophageal cancer education day hosted by a national voluntary cancer organisation that supports oesophageal cancer survivors in Ireland. Individuals were invited to express interest in participation in the study. Those who signed up to participate were contacted by a member of the research team following the education day. An appointment was made with interested participants to attend the study site to provide informed consent and complete study measures. Prior to obtaining written consent, the researcher explained the purpose of the study and answered any questions participants had about OptiMal and/or the study.

## Data Collection Methods

### Quantitative Measures

#### Multidimensional Fatigue Inventory (MFI)

The Multidimensional Fatigue Inventory (MFI) is a 20 item, self-report instrument that gathers data regarding multiple aspects of fatigue.^31^ It contains fives subscales: general fatigue, physical fatigue, reduced activities, reduced motivation and mental fatigue. Higher scores indicate higher levels of fatigue. The MFI has been validated for use with cancer patients and has demonstrated good internal consistency and construct validity.^31^

#### Hospital Anxiety and Depression Scales (HADS)

The HADS is a 14-item self-report instrument that gathers data related to possible presence of anxious or depressive states in non-psychiatric settings.^32,33^ The tool includes an anxiety and depression subscale. Higher scores indicate increased probability of clinically significant symptoms of anxiety or depression. The HADS is widely used in research and clinical practice, and has demonstrated internal consistency, and content validity when used with cancer patients in specific contexts.^33,34^

#### EQ-5D-3L

The EQ-5D-3L is a measure of health status developed by the EuroQol group.^35^ The measure consists of two parts. The first part of the measure gathers self-reported data in relation to mobility, self-care, performance of usual activities, pain and anxiety/depression. The second part of the measure consists of a visual analogue scale (EURO-VAS) where respondents rate their current health status (0–100), with higher scores indicating better health. The EQ-5D-3L has demonstrated good test-retest reliability and validity amongst cancer patients.^36^

Data were gathered at three time points during the study: prior to commencing OptiMal (T1), immediately following completion of OptiMal (T2), and three-months following completion of OptiMal (T3). T1 data were collected during the initial meeting between individual participants and a member of the research team, prior to commencement of OptiMal. Participants completed and returned T2 questionnaires in the final week of OptiMal. Three-month follow-up (T3) questionnaires were either emailed or posted to participants (with self-addressed envelope) based on participant preference.

### Quantitative Data Analysis

Statistical analysis was completed using the Statistical Package for the Social Sciences (SPSS) v.27. A cut-off point of P ≤ .05 was used to establish statistical significance in all analyses. Data were analysed using frequency and descriptive analysis to generate a demographic profile of study participants. Non-parametric statistical techniques were used given the sample size, and that not all data were normally distributed. Wilcoxon signed rank tests examined differences in MFI, HADS and EURO-VAS scores at T1, T2 and T3. Frequency analysis was used to explore trends in EQ-5D-3L scores at all time points. Chi square tests for independence examined differences in EQ-5D-3L at T1, T2 and T3. Where no differences were reported, variables were excluded from chi square tests. Fisher’s exact test values were reported given the small sample size.

### Qualitative Data Collection Methods

**Figure 2.**
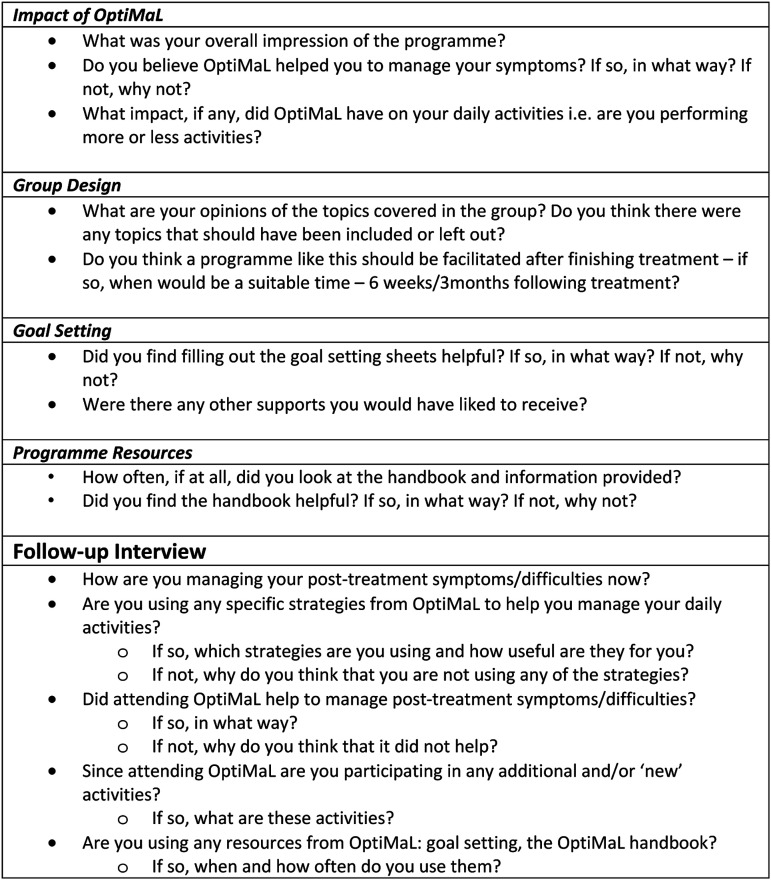
Interview guide for post-intervention focus group and follow-up individual interview.

Qualitative inquiry in this study was completed using a qualitative descriptive approach, which is characterised by adhering closely to the semantics of the data.^26^ Focus groups were carried out in the final week of OptiMal to gather participants’ experiences of attending OptiMal, the impact of OptiMal on self-management of persistent symptoms, relevance of content, and suitability of delivery format. In order to explore the longer-term impact of OptiMal on symptom management, semi-structured interviews were conducted with a convenient sample of three participants by telephone, three months following completion of OptiMal. Focus groups and interviews were carried out by senior author, DC, who has considerable experience in qualitative research. Interview guides used for the focus group and follow-up interviews were the same as those used in previous OptiMal studies (see Figure 2).

### Qualitative Data Analysis

Focus groups and individual interviews were audio-recorded and transcribed verbatim. Transcripts were then uploaded to NVivo computer software for analysis of qualitative data. Qualitative data were analysed using Braun and Clarke thematic analysis.^37^ Braun and Clark outline six stages of thematic analysis that include reading and becoming familiar with the dataset; generation of codes, reviewing and grouping codes, generation of preliminary sub-themes, and reviewing codes and sub-themes to ensure accuracy and fit with the data. The final stage is writing up and presenting the findings. Two authors (EK and DC) coded both focus groups and all follow-up interviews separately. They then met to compare their assigned codes. This process involved identifying similar codes and discussing their rationale for assigning these codes to relevant passages of text. Differences in codes were also identified. The two authors then discussed their rationale for assigning the different codes and reached agreement on whether to retain the codes and/or to rename them. Where agreement could not be reached, the third member of the team (NA) was invited to assist with reaching a consensus. Following agreement on all codes, the two authors (EK and DC) then grouped the codes into categories and created themes as recommended by Braun and Clarke.^37^ Transcripts and a summary of findings of individual interviews were returned to interviewees to enable them to remove any information given, or to amend summaries of their transcripts. None of the participants made any changes to either their transcripts or summaries.

### Ethical Considerations

Ethical approval for the study was obtained from St James’ Tallaght University Hospital Ethics Committee, reference number: 2014-12 Chairman’s Action (12). Informed consent was obtained from all participants in writing prior to commencing the study. Data were managed in line with General Data Protection Regulation (GDPR) and Health Research Regulations (2018) requirements.

## Findings

The number of attendees at the national education day was 134 individuals. However, this included people with oesophageal cancer, family members and healthcare professionals. It was not possible to specifically identify how many people with oesophageal cancer were in attendance. A total of 24 individuals expressed interest in participation in the study and provided contact details. These individuals were contacted by a member of the research team and further information on OptiMal and the study was provided. Based on the maximum number of participants for OptiMal, (8–10 individuals) 2 programmes were delivered over a 6-month period. Those who agreed to participate were asked to indicate their availability and preference for attending OptiMal.

**Figure 3.**
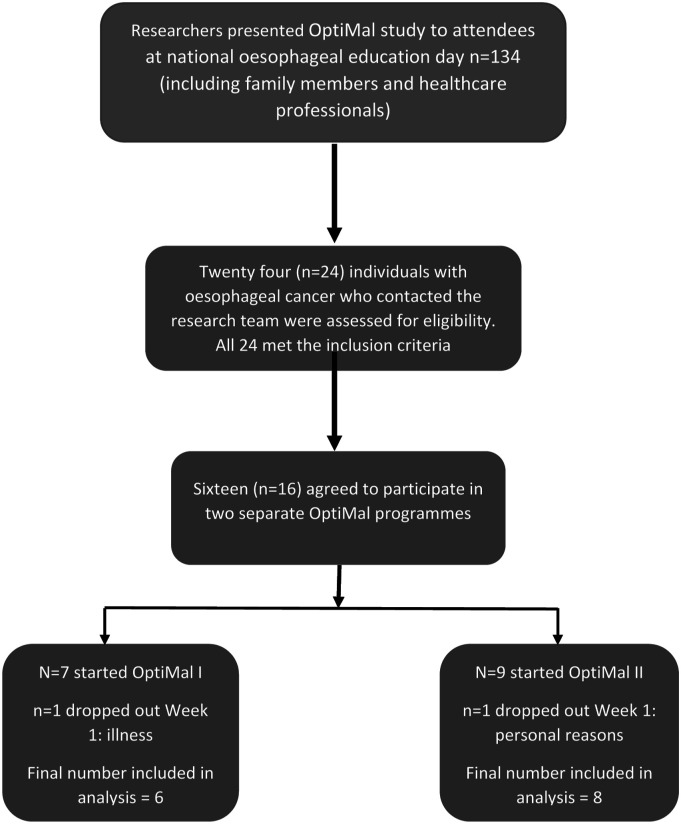
Study recruitment and attrition.

All 24 individuals who provided contact details met the eligibility criteria and of these 16 agreed to participate in the study, giving a recruitment rate of 66.8% from those who indicated interest. Reasons for non-participation included timing of the programme and travel difficulties to study centre. Seven people opted to participate in the first OptiMal programme and nine opted for the second programme. Two participants withdrew from the study after the first week of OptiMal (1 from each programme). All other participants completed the study giving a retention rate of 87.5%. Reasons for withdrawal were illness and personal reasons. Figure 3 outlines study recruitment and retention.

Fourteen participants (six from the first programme and eight from the second programme) completed baseline and follow-up outcome measures. There was a mean attendance rate at both programmes of 89.3% with eight participants (57.1%) attending all 6 weeks of OptiMal.

The majority of participants were male (n = 9, 64.3%), married (n = 11, 78.6%) and living with another person (n = 12, 85.7%). Participants’ ages ranged from 51 to 73 years at time of participation in the study, with a mean age of 61.6 years (SD 5.73). The majority of participants were not working at the time of the study (n = 9, 64.3%). See Table 1 for details of demographic characteristics.

**Table 1.** Demographic characteristics of OptiMal participants.

Demographic Profile of OptiMal Participants	n	%^a^
Sex		
Male	9	64.3
Female	5	35.7
Marital status		
Single	2	14.3
Married	11	78.6
Separated/divorced	1	7.1
Age range (years)		
50–59	5	35.71
60–69	8	57.14
70+	1	7.14
Living situation		
Living alone	2	14.3
Living with another person	12	85.7
Current employment status		
Currently employed	5	35.7
Unemployed or retired	6	42.9
Not currently working due to diagnosis/treatment	3	21.4

^a^Valid % reported only.

All participants had completed cancer treatment at time of commencing the study. The mean time since completion of cancer treatment for participants was 3.69 years (SD 4.27) with a range of 4 months to 17 years. Treatment included surgery (n = 14, 100%), chemotherapy (n = 9, 64.3%) and radiation therapy (n = 7, 50%).

### Multidimensional Fatigue Inventory

Statistically significant improvements were observed in total MFI scores from baseline (T1) to three months (T3) following participation in OptiMal, (z = −1.98, P = .05). No statistically significant differences were observed for MFI total scores between T1 and T2 (see Table 2). Individual subscales on the MFI were examined across timepoints T1 to T2, and T1 to T3. Statistically significant changes were observed in general fatigue, (z = −2.14, P = .03) and physical fatigue, between T1 and T3 (z = −2.5, P = .01). No significant differences were identified in any of the other MFI categories.

**Table 2.** Differences in patient-reported outcome measures between baseline, post-intervention and 3-month follow-up.

Measure	T1^a^ (n = 14) Median (range)	T2^a^ (n = 12) Median (Range)	P-Value (T1/T2)	Cohen’s d^b^	T3^a^ (n = 14) Median (Range)	P-Value (T1/T3)	Cohen’s d^b^
Multidimensional fatigue impact (MFI) total scores	61 (24-83)	53 (37-71)	.33	0.3	48.5 (20-80)	**.05** ^c^	0.5
^d^MFI general fatigue	14 (4-18)	12.5 (4-17)	.21	0.3	11.5 (4-16)	**.03** ^c^	0.6
^d^MFI physical fatigue	14.5 (4-18)	11.5 (8-15)	.09	0.5	10.5 (4-20)	**.01** ^c^	0.7
^d^MFI reduced activities	12.5 (4-19)	10 (7-16)	.51	0.2	9 (4-20)	.14	0.4
^d^MFI reduced motivation	8.5 (4-17)	9 (4-12)	.62	0.1	8.5 (4-16)	.75	0.1
^d^MFI Mental fatigue	11.5 (4-13)	10.5 (6-15)	.43	0.2	10 (4-13)	.2	0.3
^d^Hospital and anxiety depression scale anxiety subscale	7.5 (0-15)	6 (1-14)	0.2	0.4	6 (0-15)	.**03**^c^	0.6
^d^HADS-depression subscale	6 (0-11)	4.5 (1-9)	.14	0.4	4 (0-10)	.**005**^c^	0.8
EURO-VAS	62.5 (30-100)	70 (40-95)	.51	0.2	70 (40-90)	.28	0.3

^a^T1: baseline; T2: post-intervention; T3: 3-month follow-up. MFI: Multidimensional Fatigue Inventory; HADS: Hospital Anxiety and Depression Scale; EURO VAS: Euro QoL Visual Analogue Scale.

^b^Cohen’s d effect sizes: .2 to .50 = small to moderate; .51 to .80 = moderate to large; and >.80 = large.

^c^Statistical significance P ≤ .05.

^d^Lower scores indicate improvement.

### Hospital Anxiety and Depression Scales (HADS)

There were no statistically significant improvements in either anxiety or depression between T1 and T2 (Table 2). However, statistically significant improvements were observed in both anxiety (z = −2.19, P = .03) and depression (z = −2.83, P = .005) between T1 and T3 (Table 2).

### EQ-5D-3L

There was an overall trend of improving scores in the Euro-QoL visual analogue scale from T1 to T2 and from T1 to T3, however, this did not reach statistical significance (Table 2).

### EQ-5D-3L

The EQ-5D-3L has five health states (Mobility, Self-Care, Usual Activities, Pain and Anxiety/Depression) and three scoring categories: no problem, some problems and extreme problems. For purposes of analyses, the number of individuals reporting ‘some problems’ and ‘extreme problems’ were combined and compared with the proportion of individuals reporting ‘no problem’.

A Chi square test for independence indicated significant differences in the proportion of participants experiencing ‘some/extreme problems’ with ‘Usual Activities’ between T1 and T2, χ2 (1,n = 12) = 5.49, P = .02. This significant difference was also observed between T1 and T3: χ2 (1, n = 14) = 4.98, P = .02. Although there was a trend towards a decrease in the proportion of participants experiencing ‘some/extreme problems’ in the other two health states of ‘Pain’, and ‘Anxiety/Depression’ over time, no statistically significant differences were identified (Table 3). The categories of ‘Mobility’ and ‘Self-Care’ activities had the highest number of people reporting ‘no problem’ at the three data collection time points. No significant differences were identified in the proportion reporting ‘no problem’ and those reporting ‘some/extreme problems’ in these two health states between the three time periods.

**Table 3.** Differences in Euro QOL Health States at baseline (T1), post-intervention (T2) and 3-month follow-up (T3).

	T1 (n = 14)	T2 (n = 12)				T3 (n = 14)			
Variable	n (%)	N (%)	Chi square	*P*-value	Phi	n (%)	Chi square	*P*-value	Phi
Mobility			**.87**	**.17**	**.7**		**1.12**	**.14**	.7
No difficulties with mobilising	12 (85.7)	10 (83)				13 (93)			
Some issues with mobilising	2 (14.3)	2 (17)				1 (7)			
Self-care^a^									
No issues with performing self-care activities	14 (100)	12 (100)				14 (100)			
Some issues with performing self-care activities	0	0				0			
Usual activities			5.49	^b^ **0.02**	.9		4.98	^b^ **0.02**	.8
No issues with performing usual activities	7 (50)	7 (48)				9 (64)			
Some issues with performing usual activities	7 (50)	5 (42)				5 (36)			
Pain			0	1.0	0		1.24	.27	.5
No issues with pain	5 (35.7)	3 (25)				7 (50)			
Some pain/discomfort	9 (64.3)	6 (75)				7 (50)			
Anxiety/depression			.38	.38	.55	.4	.53	0.3	0.3
No issues with anxiety/depression	5 (35.7)	6 (50)				8 (57)			
Some anxiety/depression	9 (64.3)	6 (50)				6 (43)			

^a^Some cells of Self-Care variable contain zero, therefore, Chi Square analysis was not performed.

^b^P ≤ .05 (statistical significance).

### Qualitative Findings

In order to gather participants’ perspectives on the format, content and impact of OptiMal, two focus groups were facilitated with participants who attended the final session (i.e. Week 6) of each OptiMal programme. All five people who attended OptiMal on Week 6 participated in the first focus group, and all seven people who attended OptiMal on Week 6 participated in the second focus group. Duration of each focus group was 42 and 51 minutes, respectively. Semi-structured individual interviews were conducted with three participants three months following completion of OptiMal to explore longer-term application of the programme to daily life. Individual’s interviews were between 40 and 66 minutes in duration.

**Figure 4.**
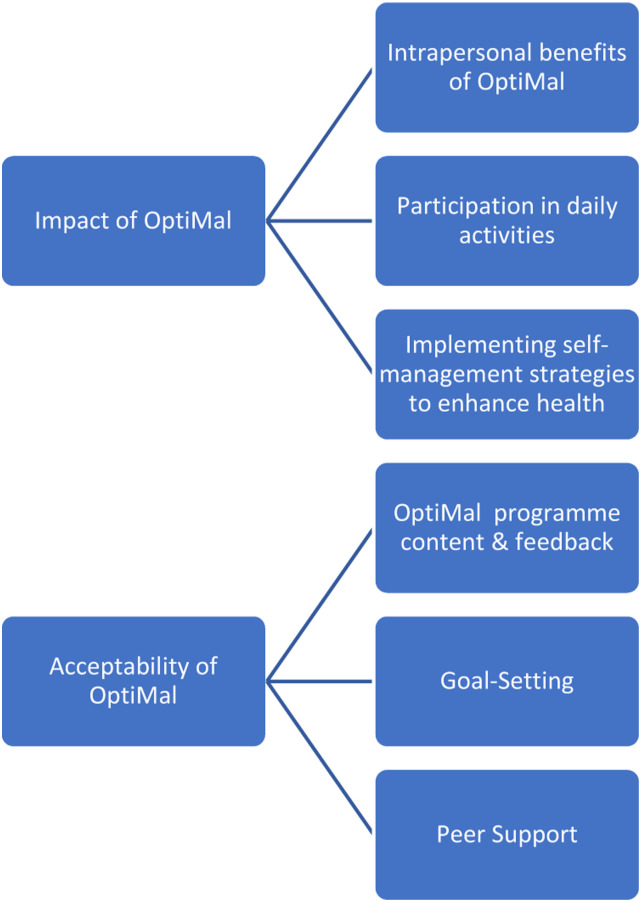
Qualitative themes and sub-themes.

On combining data from the focus groups and the three individual interviews, two themes were identified following thematic analysis: ‘Impact of OptiMal’ and ‘Acceptability of OptiMal’. In the first theme, participants identified benefits to intrapersonal skills such as increased confidence and motivation. They also identified how participation in OptiMal facilitated increased participation in valued daily activities. In the second theme, participants discussed their opinions on the acceptability of the content and format of OptiMal. Figure 4 outlines themes and sub-themes identified following data analysis.

## Theme 1: Impact of OptiMal

Three sub-themes were identified within this theme: (i) Intrapersonal benefits of the OptiMal programme which included increased confidence and empowerment (ii) Participation in daily activities where participants identified re-engaging with a range of activities following participation in OptiMal and (iii) implementing self-management strategies to improve health.

### Intrapersonal Benefits of the OptiMal Programme

In the focus groups and interviews, participants described various positive benefits of the programme in terms of psychological well-being including increased confidence, motivation, resilience, and agency and control over one’s health. Knowledge obtained during OptiMal enabled greater understanding and acceptance of participants’ health needs, and the longer-term effects of cancer and treatment. Participants also reported being more informed about how to manage their health following attendance at OptiMal:

(FG) “*I have gained an awful lot of knowledge in all areas. The knowledge I’ve gained each week, I’m going to bring that forward with me. I kind of feel I was floating along, just going through daily life. But each week I’ve gained knowledge from the course*”

One participant outlined how they believed they can manage their health effectively following participation in OptiMal and plan to continue to use goal-setting after the programme. Approximately four participants reported being more confident following OptiMal, and that the knowledge gained from OptiMal enabled them to try new exercise programmes.

Participants also discussed the importance of motivation, and many reported being more motivated to manage their health:

(P10, Int) “*I needed something to give me a kick in the bottom to get on with life as best I could.”*

### Participation in Daily Activities

Participants described returning to previously valued activities, and trialling new activities following completion of OptiMal, including leisure and work activities.

(FG): “*It set me on the road to exercise again which I hadn’t been doing*”

Another participant reported having returned to work following completion of OptiMal and how this helped him to reduce his focus on having cancer.

(P10, Int) “*Getting up in the morning, putting on my uniform, getting my lunch ready. I’m back to two days. I forget I have cancer.*”

However, finding a balance of participating in activities and managing fatigue was a challenge reported by some participants.

### Implementing Self-Management Strategies to Improve Health

Participants discussed applying their learning from OptiMal to their daily activities particularly in relation to fatigue and energy management. One interview participant summarised her key learning regarding energy management.

(P10, Int) “*Being mindful of my fatigue. Being mindful of my body and realising which time of the day is the best for me and accepting that it’s okay not to have everything done in one day*”

Fatigue management strategies, acquired during OptiMal, such as forward-planning and goal-setting were reported as facilitators for applying learning from OptiMal into everyday activities.

(FG): “*You need to plan for the different weeks your exercise, motivation, cognitive, mental… actual concrete plans*”

One participant outlined how the programme content provided insight on how to improve health and increase energy levels:

(FG) “*I’m more inspired to get my energy levels up. Whereas before the course I was just plodding along saying “Oh I’ll get better with time”. But now I’m more conscious now of all these things that are going to improve my health*”

## Theme 2: Acceptability of OptiMal

In this theme, participants discussed the relevance and usefulness of OptiMal for individuals with oesophageal cancer. They discussed their experiences of the 6-week format of OptiMal and delivery methods used by the facilitators. They also identified benefits of meeting others with oesophageal cancer and learning from each other.

### OptiMal Content and Feedback

Participants reported positive perceptions of the format and structure of OptiMal. All participants unanimously agreed that a 6-week course of 2.5 hours was an appropriate length and duration.

(FG): “*I think its fine, six weeks is long enough…and the length of sessions is fine, any longer you’d be just too tired*”

On discussing the most suitable time to attend OptiMal, eight of the 12 focus group participants reported that it would be ideal to avail of a programme like OptiMal approximately six to nine months post completion of cancer treatment.

(FG): “*I think to get on a course like this within six months of having your treatment, or your operation, would be a great idea*”

Other participants reported dissatisfaction with high cost of parking charges and recommended hosting the programme in a location that was easily accessible.

Overall, all participants reported positive experiences of OptiMal, and some participants reported that they would like to do a refresher course in the future to assist them with implementing the content into their daily lives:

(P10, Int) “*Just to say how wonderful the course was and that please God it keeps going because I felt it really did me the world of good*”

Participants were satisfied with how the group was facilitated, with ample opportunities for participants to contribute to weekly discussion topics and reported being satisfied with the relevance and usefulness of OptiMal content. They particularly valued content related to exercise, eating, looking after their mental health and well-being, and communicating with healthcare professionals and family. All participants are provided with a handbook summarising the programme content. They reported that the handbook was useful both during the group sessions and as reference material upon completion of the programme. Some participants suggested additional time for some of the topics covered such as nutrition and managing mental health, and recommended including a talk from a gastroenterologist.

### Goal-Setting

Goal-setting is an important aspect of the OptiMal programme to facilitate application of learning, with goals reviewed on a weekly basis. Examples of goals identified by participants during OptiMal included trialling fatigue management strategies, increasing physical activity levels and re-engaging with leisure and social activities such as taking holidays with family and friends.

In both the focus groups and individual interviews, participants discussed how they valued the process of setting and reviewing goals during OptiMal to maintain focus and apply their learning from the weekly OptiMal sessions:

(FG): “*I think one of the main benefits that I got from the course was setting goals each week… You had to actually keep it going and that applied to all the sessions that we had the goal*”

Another participant discussed how goal-setting motivated her to persist with her goal of travelling:

(P10, Int) “*And it did really push me. I possibly wouldn’t have done it had I not got the course to set my goal*”

### Value of Peer Support

Many participants discussed the importance of coming together as a group of oesophageal cancer survivors, and the sense of shared understanding of their experiences of receiving a cancer diagnosis, having treatment and survivorship. Participants reported reassurance in discussing symptoms with other group members.

## Discussion

Availability of self-management programmes to support oesophageal cancer survivors is limited at present.^4^ Existing programmes to support oesophageal cancer survivors tend to focus on nutrition or exercise, and evidence is needed on the impact of holistic approaches to self-management.^38^ In this study, improvements were noted in fatigue, anxiety and depression three months post-completion of the programme. Statistically significant reductions were also observed in the proportion of participants reporting difficulties with participation in usual activities three months post-completion of OptiMal. Qualitative findings lend greater insight into how participants utilised self-management strategies acquired from OptiMal, to increase their participation in daily activities and support their health and well-being.

### Acceptability of OptiMal

Participants reported mainly positive experiences of OptiMal, in terms of the content, format, resources and structure of the programme. Retention and attendance rates at each of the weekly OptiMal sessions were high, and participants shared positive feedback regarding acceptability of the programme. Some participants had to travel long distances to attend the weekly sessions but attended each week because they reported the group was important and beneficial. This supports previous research identifying a need for additional post-treatment supports for individuals with oesophageal cancer.^4^ Participants greatly valued goal-setting processes and peer support during OptiMal. They also valued sharing their experiences with other oesophageal cancer survivors, learning from each other, and reported a sense of being understood. Previous studies examining the acceptability of OptiMal for cancer survivors also reported positive experiences of the content and delivery format of the programme.^22^ Therefore, the findings of this study suggest that OptiMal is adaptable for survivors of different cancer types. The weekly content of OptiMal focuses on a range of persistent symptoms experienced by cancer survivors. These include fatigue, distress and anxiety and cognitive difficulties. Many studies have identified the impact of these symptoms across many different cancer types.^38^ OptiMal focuses on providing individuals with strategies to manage persistant symptoms and reduce their impact on daily roles and activities. Previous studies of OptiMal have demonstrated effectiveness for individuals with a range of chronic diseases.^21,23^ Therefore, as cancer is now considered a chronic disease, research on OptiMal to date indicates promising application of this self-management group-based intervention across different cancer types.

### Mood and HRQOL

The results of this study demonstrated statistically significant reduction in anxiety and depression three months following participation in OptiMal when compared to baseline scores. Puhan et al.^39^ identified minimally important clinical differences of 1.40 for the HADS depression scale and 1.32 for the HADS anxiety. Differences between baseline and both immediate post-intervention and 3-month follow-up scores achieved these differences thus demonstrating the clinical importance of OptiMal for individuals with oesophageal cancer. Similarly, a change of 3.4 was identified as clinically important in the Euro QOL EQ-VAS.^40^ This difference was observed between baseline and the two follow-up data collection periods.

Previous studies also reported significant decreases in anxiety and depression scores in oesophageal cancer survivors following completion of a nursing led rehabilitation intervention.^18,19^ Chen et al. reported lower scores on anxiety and depression subscales as measured by the HADS in oesophageal cancer survivors following completion of a rehabilitation programme addressing quality of life, sleep and well-being, but this did not reach statistical significance.^19^ Housman and colleagues suggested various reasons for psychological distress following oesophageal cancer diagnosis and treatment including the trauma of diagnosis, the intensity of oesophageal cancer treatment, risk of adverse outcomes or post-surgical complications, need to adjust to a new way of life following treatment and possible biological predispositions to conditions such as anxiety or depression.^13^ These range of difficulties support the need for post-treatment interventions to reduce the impact of psychological distress.

In this study, participants reported positive perceptions of OptiMal in relation to motivation, confidence, resilience and increased sense of agency over their health. They described greater understanding of their own health needs and increased confidence in managing their health after attending the six-week programme. This is an important finding as numerous studies reported that oesophageal cancer survivors may find establishing and maintaining agency over daily life challenging in the period following completion of cancer treatment and that individuals may defer important decision-making to ‘expert’ medical professionals.^6,8,12^ Other studies have reported that increased confidence is a significant predictor of quality of life in individuals with oesphageal cancer.^41^

Oncology teams are not always well equipped to meet the psychological or emotional support needs of oesophageal cancer survivors.^12^ This is concerning given that anxiety and/or depression are linked to poorer health outcomes.^13,42^ However, the findings from the current study may yield some promising results for the impact of self-management interventions with regards to mood and well-being of oesophageal cancer survivors.

Fatigue impacts on ability to participate in daily life activities, is common amongst oesophageal cancer survivors, and may persist for an extended period following completion of treatment.^7^ It is interesting to note, however, that many studies examining interventions to support oesophageal cancer survivors do not examine changes in fatigue over time. The findings of this study indicated an improvement in participants’ fatigue with significant reductions in general and physical fatigue three months following completion of OptiMal. Study participants discussed how they used energy conservation strategies and had a greater understanding of the importance of prioritising self-care following the programme, which supported them in implementing fatigue management strategies. Zhang et al reported improved ability of oesophageal cancer survivors to engage in self-care activities, following an individualised nursing led rehabilitation programme.^18^ The current study sought to evaluate a group-based self-management programme. Therefore, in considering the combined findings of quantitative and qualitative analysis, OptiMal offers some initial promising results for future evaluations of fatigue management for oesophageal cancer survivors.

### Participation in Daily Activities

In qualitative focus groups and follow-up interviews, participants discussed engaging in new or previously valued meaningful activities such as work and leisure following participation in OptiMal. These findings are important given that Larsen et al reported that oesophageal cancer treatment necessitated ‘putting life on hold’, and caused major disruption to daily activities, roles and routines.^12^ Existing literature shows that oesophageal cancer survivors may find establishing new routines challenging following oesophageal cancer surgery,^4^ and therefore the findings of this current study that participants experienced less difficulty with performance of usual activities over time is important and supports further evaluation of OptiMal for oesophageal cancer survivors. van Deudekom and colleagues recommended that approaches that focus on supporting participation in daily life activities could be useful to improve quality of life outcomes in oesophageal cancer survivors.^43^ HRQoL scores improved following completion of OptiMal, and remained at similar improved levels over time, but no statistically significant differences were identified. These findings are interesting and warrant further investigation in future studies with larger sample sizes. Other studies examining interventions to improve quality of life of oesophageal cancer survivors did not observe statistically significant improvements in global HRQoL, but did identify significant changes in domains of HRQoL, including pain, insomnia and financial difficulties.^19^ It is important to include robust measures of HRQoL in future studies examining outcomes of programmes to support oesophageal cancer survivors.

### Limitations

A sample size was not calculated for this study and there was a lack of a control group for the intervention which precludes analysis of the effectiveness of the OptiMal programme. The study sample was small, however, the study design incorporates a mixed methodology, with both quantitative and qualitative data to lend a rich insight into the outcomes and experiences of the programme participants. Three months follow-up interviews were completed with only three OptiMal participants. Additional interviews would lend greater insight into the longer-term experiences of participants upon completion of the OptiMal programme.

The EORTC Quality of Life Questionnaire-Oesophageal Cancer Module (EORTC QLQ-. OES18)^44^ is a specific measure of symptoms and emotional problems related to oesophageal cancer. However, in all other studies of OptiMal, the EQ-5D-3L was used to measure quality of life. Therefore, in order to maintain consistency with previous studies of OptiMal, the EQ-5D-3L was used in this study. However, in future studies, the EORTC Quality of Life Questionnaire – Oesophageal Cancer Module (EORTC QLQ-OES18) could be included as a validated measure of quality of life of individuals with oesophageal cancer.

## Conclusion

The findings of this feasibility study indicate feasibility and acceptability of OptiMal for oesophageal cancer survivors. The content, format and delivery were reported as relevant and appropriate for individuals following completion of their cancer treatment and retention and adherence rates were high. Improvements in fatigue, participation in daily activities, anxiety and depression three- months following completion of OptiMal indicate potential effectiveness of this intervention. Previous studies examining the outcomes of OptiMal also identified increased frequency of activity participation, activity performance, satisfaction, self-efficacy, independence in daily activities and quality of life following participation.^24^ Availability of self-management programmes for this population is limited at present, therefore, the findings of this study indicate promising outcomes for self-management interventions for this population. However, future research is required using robust, controlled study designs with a larger sample size in order to investigate the efficacy of OptiMal for oesophageal cancer survivors.
